# Midgut Neuroendocrine Tumours: Are They at Higher Risk of Anastomotic Leak Than Midgut Adenocarcinomas? A Case–Control Study

**DOI:** 10.1002/cnr2.70532

**Published:** 2026-04-15

**Authors:** Sara‐Jane Horne, Mark Coleman, Jon Bishop

**Affiliations:** ^1^ University Hospitals Plymouth Plymouth UK; ^2^ Academic Department of Military Surgery and Trauma UK; ^3^ University of Birmingham Birmingham UK

**Keywords:** anastomotic leak, neuroendocrine neoplasia, neuroendocrine tumours

## Abstract

**Background:**

The incidence of neuroendocrine tumours is increasing; they are now the most prevalent small bowel malignancy.

**Aims:**

To identify whether primary anastomoses following resections of midgut neuroendocrine tumours (NETs) are at higher risk of anastomotic leak than primary anastomoses following midgut adenocarcinoma resections.

**Methods and Results:**

Retrospective analysis of all cases of midgut NET resections and primary anastomoses at a tertiary university hospital in the UK 2019–2024, comparing these to controls of midgut adenocarcinoma resections and primary anastomoses at the same hospital during the same time period. 35 NET cases were matched with 35 adenocarcinoma controls, with anastomotic leaks in 4 (11.4%) cases and 2 (5.7%) controls. The morbidity rate was slightly higher in the NET cohort, occurring in 17 (49%) cases compared to 15 (43%) in the adenocarcinoma controls. However, the proportion of severe complications in the adenocarcinoma controls was higher. Neither of the cohorts had mortalities within 30 days of surgery. Overall all‐cause mortality rates and median survival post‐operatively were comparable between the two cohorts.

**Conclusion:**

NETs may be an independent risk factor for anastomotic leak rates; however, further evidence with larger cohorts of patients is required. Due to the low incidence of midgut NETs and midgut adenocarcinomas, a national collaborative is recommended.

## 1 Introduction

Neuroendocrine neoplasms (NENs) are sub‐divided into well‐differentiated neuroendocrine tumours (NETs) and poorly‐differentiated neuroendocrine carcinomas (NECs). NETs develop more frequently in the gastroenteropancreatic (GEP) tract and NECs mostly arise in the lung [1]. GEP NENs will be referred to as NETs from here on.

NENs have historically been rare cancers, but their incidence has significantly increased over the last few decades, with rates reported to be at 371%–400% since 1978 [2, 3]. NENs are most common in the lungs and small bowel [4, 5], which is the site of approximately 20% of all NENs [6]. NETs are the most common malignancy of the small bowel and account for 37% of small bowel malignancies, having surpassed adenocarcinomas [5, 7]. Small bowel NETs are the most likely to develop distant metastases [6], with rates of 35% reported [8]. Current guidelines advise consideration of primary resection, even if disease has metastasised to local lymph nodes [9, 10].

It was anecdotally noted by the General Surgical Department at University Hospitals Plymouth (UHP), a tertiary university hospital in the United Kingdom, that primary anastomoses following NET resections might be at higher risk of anastomotic leak than primary anastomoses following adenocarcinoma resections. Anastomotic leak in this study is defined as failure of a gastrointestinal (GI) tract anastomosis to heal, causing either localised or generalised contamination of the abdomen with GI tract contents and confirmed on computed tomography (CT) cross‐sectional imaging.

Carcinoid tumours are the most common type of GI NET, which was in keeping with our data (29 (83%) NET cases), and the most common hormone secreted by carcinoid tumours is serotonin or 5‐hydroxytryptamine (5‐HT) [11]. Serotonin can cause either vasodilation or vasoconstriction, depending on the integrity of the vessel endothelium, sympathetic tone, local modulating factors such as partial pressure of oxygen and temperature, and chronic modulating factors such as blood pressure [12]. Therefore, it is difficult to hypothesise what the overall effect of a serotonin‐producing NET would be on vasculature and healing. Another hypothesis is that NETs involving the mesenteric vasculature can cause mesenteric fibrosis and/or ischaemia [13], which would suggest that anastomoses following NET resections might be less likely to heal.

Review of published literature found no studies looking at NETs as a risk factor for anastomotic leak. Due to the rarity of NETs, midgut NETs were selected as the most prevalent site of GI NETs in order to increase case numbers. The consensus guidelines from the North American Neuroendocrine Tumour Society (NANETS) also reference this method of categorisation using embryological origins of the GI tract [6, 7, 14], therefore it was decided to look at cases of NETs affecting the embryological ‘midgut’, from the second part of the duodenum to two thirds along the transverse colon. This included various bowel types, which is why NETs cases were matched as closely as possible by anatomy in the first instance with the adenocarcinoma cases.

## 2 Methods

**FIGURE 1:**
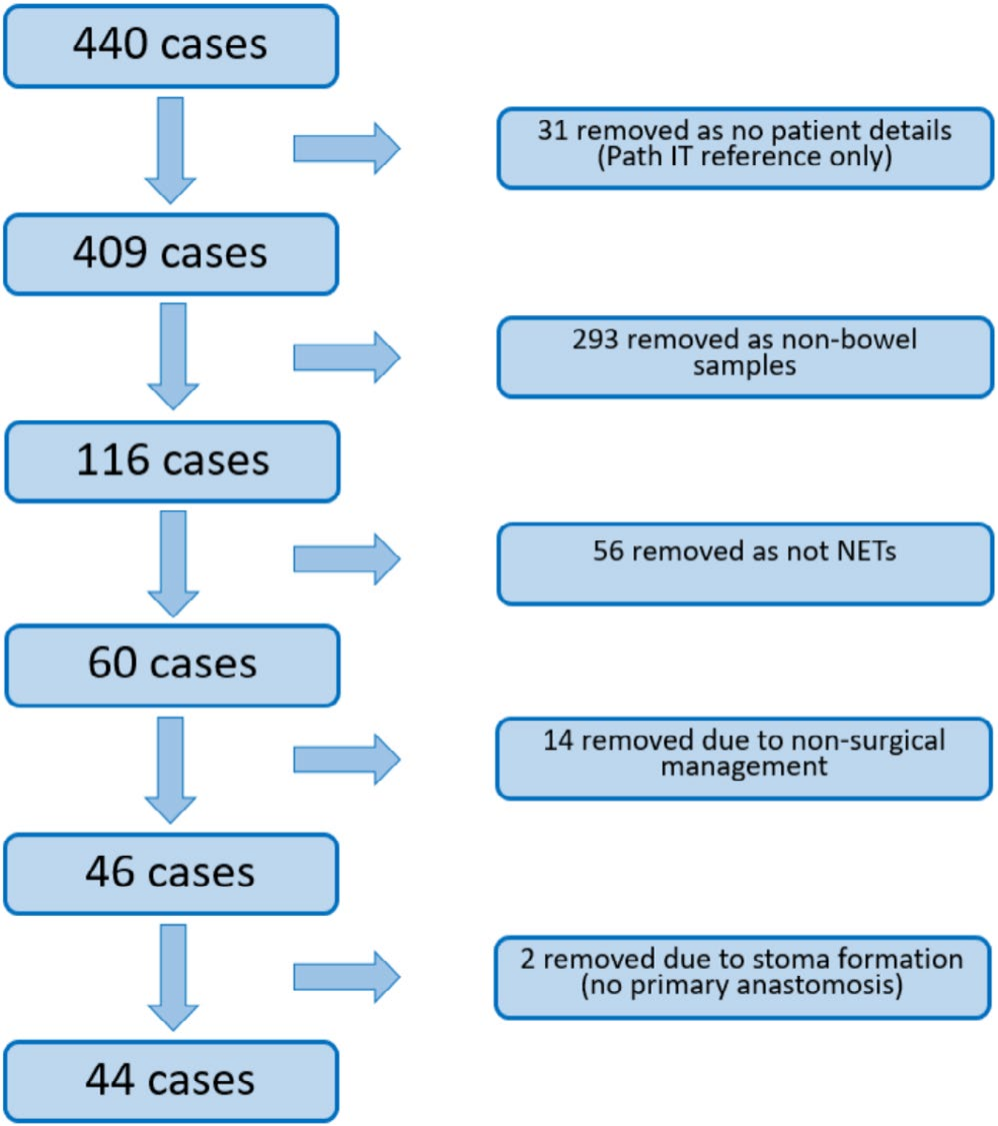
Flowchart demonstrating selection of NETs cases.

UHP's ‘Pathology IT’ database was used to search for all histology specimens analysed at UHP between 01 March 2019 and 01 March 2024 and coded as NETs, see Figure 1.

**FIGURE 2:**
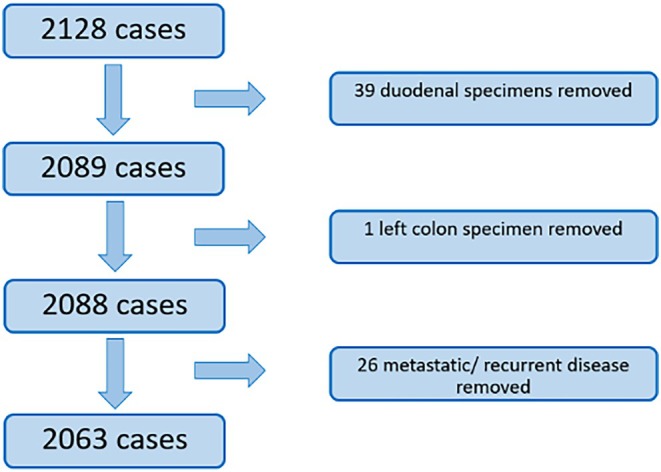
Flowchart demonstrating selection of adenocarcinoma cases.

The same database was used to search for all small bowel, caecum, ascending colon and/or transverse colon histology specimens analysed at UHP between 01 March 2019 and 01 March 2024 and coded as adenocarcinomas, see Figure 2.

One control case from the adenocarcinoma cohort was selected for each NET case according to the variables shown in Table 1, with the variables prioritised in the order shown. Matching the cases in a set order of variables helped to reduce potential selection bias associated with a retrospective analysis.

**TABLE 1:** The variables used for controlling the NET cases with adenocarcinoma cases, and the order in which variables were prioritised.

Variable	Incidence in NETs cohort (*n* = 35)	Incidence in adenocarcinoma cohort (*n* = 35)
Type of resection		
Duodenal‐jejunal flexure resection	0	1
Small bowel resection (SBR)	8	8
SBR + appendicectomy	2	0
SBR + right hemicolectomy	5	1
Right hemicolectomy	19	22
Right hemicolectomy + ‘other’^a^	1	3
Median age at operation	66 (IQR 14)	67 (IQR 15)
ASA		
1–2	17	16
3–4	18	19
Male sex	16	16
Site(s) of tumour		
Duodenal‐jejunal flexure	0	1
Small bowel	27	11
Appendix	5	4
Caecum	7	10
Right colon	0	8
Transverse colon	1	2
Open/minimally invasive surgery		
Open	18	14
Laparoscopic	15	17
Robotic	2	3
Unknown	0	1
Smoking status		
Current	4	1
Former	11	12
Never smoked	14	15
Unknown	6	7
Previous radiotherapy	0	1
Unknown	1	1
Oral steroids < 3 months pre‐operatively	1	1
Unknown	1	2
Excessive alcohol consumption	3	2
Unknown	11	17
BMI > 30	8	11
Unknown	11	13
Pre‐operative tumour markers < 2 months	16	14
Pre‐operative raised hormonal markers (raised chromogranin A/B, raised 24‐h urinary 5‐HIAA excretion/volume)	8	
Positive immunohistochemistry	14	
Pre‐operative renal impairment		
CKD 1–2	18	14
CKD 3–4	2	4
Pre‐operative hepatic impairment	0	2

^a^ Omentectomy (*n* = 1), Meckel's diverticulectomy (*n* = 1), lap hysterectomy + R salpingo‐oophorectomy (*n* = 1), SBR + wedge gastrectomy (*n* = 1).

The majority of cases (34 (97%)) were matched according to type of resection, with the only outlier being the duodenal‐jejunal flexure resection case being controlled with a small bowel resection case. Similarly, 34 (97%) cases were matched to within 10 years of age at operation, except one case with an age difference of 12 years. The mean age difference was 4.4 years (SD 3.2). Approximately one third (12 (34%)) of case–controls were not matched in the ASA categories of 1–2 and 3–4, but only 1 case–control had a difference in ASA score of more than 1 point.

Most cases were matched for sex (25 (71%)), site of tumour (25 (71%)) and for open versus laparoscopic or robotic approaches (categorising laparoscopic and robotic approaches together) (24 (69%)).

Smoking status was the same in 22 (63%) case–control pairs, though in 6 (19%) pairs smoking status was not documented. Alcohol consumption was only documented in 23 (66%) case–control pairs, but 20 (87%) of these were concordant. Contemporaneous pre‐operative BMIs were also difficult to confirm with only 48 (69%) cases and controls being recorded, though the discrepancy rate between cases and controls being obese or not obese was only 11%. However, as BMI is a continuum, use of a binary threshold of 30 kg/m^2^ will not accurately represent how closely the cases and their controls were matched in terms of BMI.

Only 1 case had previously undergone radiotherapy so it was not possible to match on this variable. The two cases who had been treated with oral steroids within 3 months of surgery were not matched due to other variables being prioritised, but neither of these cases nor their controls had the complication of anastomotic leak.

Only 28 (40%) cases had pre‐operative tumour markers; the NET cases were predominantly tested for chromogranin A and B, though 6 (17%) were also tested for CEA (half of these were raised). Of the NET cases, 14 (40%) were tested for hormonal markers (chromogranin A, chromogranin B, 24‐h urinary 5‐HIAA excretion and 24‐h urinary 5‐HIAA volume). Of the NET cases tested, 8 (57%) had raised hormonal markers (23% of all NET cases). Among the NET cases, 14 (40%) had positive immunohistochemistry, 4 (29%) of these were also tested for hormonal markers, and all 4 cases had raised hormonal markers. Of the 4 (11%) NET cases complicated by anastomotic leak, 2 had positive immunohistochemistry, one of which had positive hormonal markers as well.

On pre‐operative renal function tests, 23 (66%) cases were within 1 CKD category of their controls, and only 2 adenocarcinoma cases showed hepatic impairment on pre‐operative liver function tests.

The data were analysed with log‐binomial regression models to produce estimated relative risk ratios and associated 95% confidence intervals. A regression‐based approach was used rather than matched‐pair statistics due to the large number of variables looked at and the inability to match all of these variables for the cases and controls due to the low sample size. The initial analysis adjusted only for participant group (case versus control), with further models then adjusting for a single additional covariate according to the variables in Table 1 (age, ASA grade, smoking status, alcohol intake, obesity status, pre‐op radiotherapy, pre‐op steroids). Age was included as a continuous variable, all other covariates were categorical.

## 3 Results

Table 2 shows the summary of outcomes. Of the 440 NET cases returned on the initial search, 44 were eligible for inclusion. However, only 35 of these could be matched with control cases from the 2063 eligible adenocarcinoma histology specimens analysed at UHP between March 2019 and March 2024, so 9 unmatched NET cases were excluded. This was due to being unable to match the types of resection, which was the first priority variable in Table 1. In the NET cohort, four patients (11.4%) had an anastomotic leak compared to two (5.7%) in the adenocarcinoma cohort (relative risk ratio: 2.00, 95% confidence interval: 0.42 to 13.78).

**TABLE 2:** Table of outcomes.

	Anastomotic leak rate	Clavien‐Dindo I‐II	Clavien‐Dindo III‐IV	All‐cause mortality	Median survival of mortalities (days)
Neuroendocrine tumour cases	4 (11.4%)	13 (37%)	4 (11%)	5 (14%)	487 (IQR 415.5)
Adenocarcinoma controls	2 (5.7%)	9 (26%)	6 (17%)	4 (11%)	494 (IQR 674.5)

The estimated relative risk ratios for leak rates had high levels of uncertainty in all fitted models, and this study was severely underpowered to detect any clinically meaningful difference in leak rates between the groups. As an example, assume the observed difference of 5.4% in anastomotic leak rates between groups was deemed to be clinically important. Then, to have 80% power to detect this difference, assuming rates of 11.4% and 5.7% in the NETs and adenocarcinoma cohorts respectively, we would require outcome data on 748 patients (374 in each group). Studies would require still larger sample sizes if smaller differences in anastomotic leak rates were deemed to be clinically important, or higher power was required.

Overall morbidity occurred in 17 (49%) patients in the NET cohort, with 13 (76%) of these having complications of Grade I–II on the Clavien‐Dindo classification and 4 (24%) of Grade III–IV. The overall morbidity was slightly lower in the adenocarcinoma cohort, occurring in 15 (43%) patients, though the proportion of serious complications was relatively higher, with 10 (60%) having complications of Grade I–II and 5 (40%) of Grade III–IV.

Neither of the cohorts recorded any mortalities within 30 days of surgery. Overall, all‐cause mortality in the NET cohort was 5 (14%) patients, with a mean survival of 387.6 days post‐operatively, and only one of these cases died within 360 days of surgery. In the adenocarcinoma cohort, 4 (11%) experienced all‐cause mortality and had a mean survival of 674.5 days post‐operatively.

Of the four patients in the NET cohort who had anastomotic leaks, three of these underwent right hemicolectomy and one underwent small bowel resection. Of the two patients in the adenocarcinoma cohort who had anastomotic leaks, one underwent right hemicolectomy and one underwent small bowel resection.

## 4 Discussion

### 4.1 Key Results

Our data indicate that primary anastomoses following NET resections could be at higher risk of anastomotic leak than primary anastomoses following adenocarcinoma resections, and that NETs may be an independent risk factor for anastomotic leaks.

### 4.2 Limitations

However, our data are limited by low case numbers. This is largely due to the low incidence of midgut NETs (0.32–1.46 per 100 000) [2, 7, 9] and the now even lower incidence of midgut adenocarcinomas (1.5–3.0 per 100 000) [7, 15]; 20.5% of NET cases were excluded due to a lack of adenocarcinoma controls.

To sample a cohort large enough for an adequately powered study, the timeframe and number of centres where these patients were treated would need to be expanded. Expansion of the timeframe for data collection might introduce bias due to the rapid evolution of surgical techniques and equipment, and the resulting reduction in complication rates over time. This potential variable could be reduced by still controlling each case within a shorter timeframe. Involving other centres to increase the cohort size might also introduce bias due to variation in local practices and surgical technique. However, with the National Institute for Health and Care Excellence (NICE), NANETS [9] and ENETS [10] guidelines, surgical practice in the United Kingdom (UK) should be standardised with minimal regional variation. Also, the more centres involved, the less significant this bias would be. If data were to be collected internationally, the potential bias from varying patient populations would also need to be considered.

Variables within the surgical care provided at UHP were not looked at, such as surgeon seniority, subspecialty or number of operating surgeons, all of which may have influenced the risk of anastomotic leak.

Each case control was reviewed systematically according to the variables in Table 1; however, the relative contribution of each variable to the matching process was decided by the authors due to a lack of evidence on the strength of association each variable had with the risk of anastomotic leak. These variables were selected based on recognised risk factors for anastomotic leaks in colon cancer [16]. Polcz et al. reported specific variables negatively associated with survival in metastatic midgut NETs (not necessarily associated with anastomotic leak rates), which included colon and appendiceal primaries, comorbidities (ASA used as an indication of this) and increasing age [8].

### 4.3 Comparison to Literature

Polcz et al. also reported that 76% of their cases were jejuno‐ileal, which is consistent with the 77% (‘small bowel’) in our data, and that 24% affected the right colon or appendix, which is lower than the 34% seen in our data [8]. The median age in our NETs cohort of 66 years is also consistent with the literature, with Watzka et al. finding a median age of 62 [17].

Open versus laparoscopic or robotic surgery was one of the other variables considered when selecting case‐controls. Both NANETS [9] and ENETs [10] guidelines recommend exploratory laparotomy, where feasible, as the gold standard over imaging modalities for NETs [18]. However, minimally invasive surgery is now globally recognised as superior for overall patient outcomes. For reducing anastomotic leak rates, the literature also supports non‐inferiority/superiority of laparoscopic GI resections [17, 19, 20, 21], compared to open surgery and there is some evidence to support laparoscopic resection of NETs [18, 22, 23].

## 5 Conclusions

The data on anastomotic leak rates of NETs are sparse, and these authors could find no studies investigating NETs as a risk factor for anastomotic leaks. This study indicates that primary anastomoses following NET resections may be at higher risk of anastomotic leak than primary anastomoses following adenocarcinoma resections, however, the data are limited by the low case number of 70 patients in total. Sample size calculations indicate that over 700 patients would be required to have 80% power to detect even large effect sizes (e.g., relative risk ratio of 2); a national or international collaborative is recommended for achieving this.

This study suggests that general surgeons undertaking resection and primary anastomosis of NETs should be aware that there may be an increased risk of anastomotic leak in these cases relative to adenocarcinomas; however, our data were not statistically significant to validate this nor to indicate the relative risk. If this hypothesis were to be proven with a larger study, it is unlikely that a relative risk ratio of less than 1.1 would alter surgical decision‐making looking at NETs as an independent risk factor for anastomotic leak, but it would be more likely that clinicians would take this into consideration alongside all the other variables associated with increased risk of anastomotic leak [16].

Up to 35% of midgut NETs may present with an acute intra‐abdominal complication, such as obstruction, perforation, bleeding or ischaemia [24], which means that any general surgeon on call might need to operate on a NET, even if it is not their sub‐specialty. This statistic also indicates that NETs should be included in the curriculum of general surgical trainees; however, they are not explicitly mentioned in the current UK Intercollegiate Curriculum Surgical Programme (ISCP) curriculum for General Surgery. NETs may be included in the colorectal, rectal and anal neoplasia mentioned, but they are most common in the small bowel. Gastrointestinal stromal tumours (GISTs) and lymphoma are specifically mentioned, even though both of these types of neoplasia are rarer than NETs in the small bowel [25].

## Author Contributions

**Sara‐Jane Horne:** conceived the study, undertook the data collection and wrote the manuscript. **Mark Coleman:** methodology, writing – review and editing, supervision. **Jon Bishop:** writing – review and editing, formal analysis, data curation.

## Funding

The authors have nothing to report.

## Ethics Statement

This retrospective cohort study used routinely collected patient data from University Hospitals Plymouth. The dataset was anonymised by a member of the clinical care team (SJH) prior to analysis. In accordance with Health Research Authority guidance, research using fully anonymised data does not require NHS Research Ethics Committee approval. The project was registered with the University Hospitals Plymouth Research & Development department.

## Consent

All data were fully anonymised prior to analysis and had been collected as part of routine clinical practice; therefore patient consent was not required.

## Conflicts of Interest

The authors declare no conflicts of interest.

## Data Availability

The data supporting the findings of this study contain anonymised patient information and are therefore not publicly available due to data protection considerations. Data may be made available from the corresponding author upon reasonable request, subject to appropriate approvals.
